# State Examinations for License, New York

**Published:** 1894-12

**Authors:** 


### STATE EXAMINATIONS FOR LICENSE, NEW YORK.

FROM advance sheets of the report of the regents of the University of the State of New York for 1894, we are permitted to extract the following statistics, that we think will prove of interest to our readers :

| | Examinations. | | Candidates. | | Examined. | | Accepted. | | Rejected. | | Per cent. Accepted. | | Per cent. Rejected. | |
|---|---|---|---|---|---|---|---|---|---|---|---|---|---|---|
| | 1893 | 1894. | 1893 | 1894. | 1893. | 1894. | 1893. | 1894. | 1893. | 1894. | 1893. | 1894. | 1893. | 1894. |
| State Board.... | 8 | 6 | 296 | 427 | 267 | 390 | 247 | 311 | 20 | 79 | 92.5 | 79.7 | 7.4 | 20.2 |
| Homeo. Board. | 8 | 6 | 23 | 56 | 21 | 51 | 19 | 44 | 2 | 7 | 90.4 | 86.2 | 9.5 | 13.7 |
| Eclectic Board. | 8 | 6 | 7 | 6 | 7 | 4 | 5 | 2 | 2 | 2 | 71.4 | 50. | 28.5 | 50. |
| Total........ | 8 | 6 | 326 | 489 | 295 | 445 *a* | 271 | 357 *a* | 24 | 88 *a* | | | | |

*a.* These totals from September 1, 1893, to July 31, 1894, include two candidates who paid three times and are, therefore, counted three times, and seven candidates who paid twice and are, therefore, counted twice ; two candidates whose papers were not examined because of attempted fraud, and one eclectic candidate who has not yet completed his examinations.

The number of the last license issued in 1894 was 687.

### LAW AND MEDICAL STUDENT EXAMINATIONS, AUGUST 1, 1893—AUGUST 1, 1894.

| | |
|---|---|
| Examinations | 6 |
| Answer papers written | 13,968 |
| Answer papers accepted | 9,330 |
| Answer papers rejected | 4,638 |
| Per cent. of papers accepted | 66.8 |
| Per cent. of papers rejected | 33.2 |
| Law student certificates issued | 475 |
| Law student certificates issued on examination | 424 |
| Law student certificates issued, equivalents | 51 |
| Medical student certificates issued | 947 |
| Medical student certificates issued on examination | 395 |
| Medical student certificates issued, equivalents | 552 |
| Per cent. of law student certificates issued on equivalents | 10.7 |

| | |
|---|---|
| Per cent. of medical student certificates issued on equivalents.. | 58.2 |
| Academy equivalent certificates issued........................ | 259 |
| Academy equivalent certificates issued on examination........ | 24 |
| Academy equivalent certificates issued on equivalents......... | 235 |
| Per cent. Academy equivalent certificates issued on equivalents. | 90.7 |

